# Photo-tautomerization of acetaldehyde as a photochemical source of formic acid in the troposphere

**DOI:** 10.1038/s41467-018-04824-2

**Published:** 2018-07-03

**Authors:** Miranda F. Shaw, Bálint Sztáray, Lisa K. Whalley, Dwayne E. Heard, Dylan B. Millet, Meredith J. T. Jordan, David L. Osborn, Scott H. Kable

**Affiliations:** 1 School of Chemistry, University of Sydney, Sydney, NSW 2006 Australia; 2 Department of Chemistry, University of the Pacific, Stockton, CA 95211 USA; 3 School of Chemistry and National Centre for Atmospheric Science, University of Leeds, Leeds, LS2 9JT UK; 4 Department of Soil, Water, and Climate, University of Minnesota, Minneapolis–Saint Paul, MN 55108 USA; 5 Combustion Research Facility, Sandia National Laboratories, Livermore, CA 94551 USA; 6 School of Chemistry, University of New South Wales, Sydney, NSW 2052 Australia

## Abstract

Organic acids play a key role in the troposphere, contributing to atmospheric aqueous-phase chemistry, aerosol formation, and precipitation acidity. Atmospheric models currently account for less than half the observed, globally averaged formic acid loading. Here we report that acetaldehyde photo-tautomerizes to vinyl alcohol under atmospherically relevant pressures of nitrogen, in the actinic wavelength range, *λ* = 300–330 nm, with measured quantum yields of 2–25%. Recent theoretical kinetics studies show hydroxyl-initiated oxidation of vinyl alcohol produces formic acid. Adding these pathways to an atmospheric chemistry box model (Master Chemical Mechanism) demonstrates increased formic acid concentrations by a factor of ~1.7 in the polluted troposphere and a factor of ~3 under pristine conditions. Incorporating this mechanism into the GEOS-Chem 3D global chemical transport model reveals an estimated 7% contribution to worldwide formic acid production, with up to 60% of the total modeled formic acid production over oceans arising from photo-tautomerization.

## Abstract (web summary)

The concentration of formic acid in Earth’s atmosphere is under-predicted by atmospheric models. Here the authors show that acetaldehyde photo-tautomerizes to vinyl alcohol under tropospheric conditions, with subsequent oxidation via OH radicals supplying up to 60% of total modeled formic acid production over oceans.

## Introduction

Organic acids are ubiquitous in Earth’s atmosphere and are detected in urban, rural, polar, tropical, and marine environments. They are present in the gas phase, and in clouds and aerosols where they substantially impact rainwater acidity^1,2^ and aqueous-phase chemistry. The high oxygen content of organic acids dramatically reduces volatility compared to less oxidized molecules of similar molar mass and the larger organic acids are believed to be key species in the nucleation and growth of atmospheric particles^3–6^, which have significant impacts on human health, local pollution, and global climate^7^.

Over the last decade, improved measurement techniques^8,9^ have repeatedly shown significantly higher organic acid concentrations than predicted by atmospheric models^10^. This disagreement, which is particularly marked in the daytime, implies missing or underestimated photochemical source terms, or overestimated organic acid sinks. The smallest organic acids, formic (HCOOH, FA) and acetic (CH_3_COOH, AA) acid, dominate global tropospheric organic acids, comprising >60% of free acidity in pristine rainwater and >30% in polluted areas^10^. Although fossil fuel combustion emits organic acids directly, ^14^C/^12^C isotope ratios show atmospheric HCOOH is mainly modern carbon, with fossil fuel sources making only minor contributions^11^. The majority of atmospheric organic acids are believed to be produced via photochemical oxidation of biogenic and anthropogenic volatile organic compounds (VOCs). The largest global source of FA is thought to be photochemical production, though the magnitude of this is highly uncertain^10^. Plants emit FA directly^12^ but also reabsorb it, complicating determinations of the net effect, although Schobesberger et al. report recently that forests might be a net source^13^. Forest fires^14,15^ and photochemical oxidation of organic aerosols^16^ have also been proposed as important sources of FA. A recent study^10^ noted FA concentrations in Earth’s boundary layer of several parts per billion, which are 2–3 times larger than predicted from currently known production and consumption pathways. They concluded that photochemical biogenic sources are dominant but currently underestimated, and that there must be widespread sources of FA from a variety of precursor species.

One potential source of FA is the photo-tautomerization of acetaldehyde (CH_3_CHO, AC) to its enol form, vinyl alcohol (CH_2_=CHOH, VA). Archibald et al.^17^ first suggested that enols, emitted directly from combustion sources, would oxidize in the atmosphere to produce organic acids. Andrews^18^ subsequently postulated, based on isotopic scrambling studies of AC, that even larger sources of atmospheric enols could arise from sunlight-driven tautomerization of carbonyl compounds. Clubb^19^ provided the first experimental evidence of AC → VA photo-tautomerization, albeit for neat AC at low pressure under non-atmospheric conditions. Da Silva^20^ then used electronic structure and master equation calculations to conclude that oxidation of VA by the hydroxyl radical (OH) is a fast reaction that produces mainly FA. Given this background, Millet^10^ evaluated the global importance of the keto-enol photo-tautomerization hypothesis, and concluded that this new AC photo-tautomerization mechanism constitutes ~15% of global FA sources, but suggested its impact is limited because FA itself catalyzes the reverse tautomerization, VA → AC, effectively buffering its own production^21^. However, Peeters^22^ found the rate of gas-phase reverse tautomerization to be six orders of magnitude slower than the value reported by da Silva^21^, which was used previously by Millet et al.^10^, and concluded that gas-phase reverse tautomerization was unimportant in the atmosphere^22^.

In this contribution, we report direct experimental evidence under atmospheric conditions that AC does indeed photo-tautomerize to VA. We provide pressure- and wavelength-dependent quantum yields for VA production in up to 1 atm of N_2_ in the actinic wavelength range of *λ* = 300–330 nm. To evaluate the impact on FA sources, we implement photo-tautomerization, VA oxidation, and acid-catalyzed gas-phase reverse tautomerization into two atmospheric models: (i) a detailed chemistry box model which uses the Master Chemical Mechanism^23^, and (ii) the GEOS-Chem 3D chemical transport model (CTM)^24^. These simulations allow us to determine, on a global basis, that the photo-tautomerization mechanism contributes an estimated 7% to worldwide formic acid production, and up to 60% of the total modeled formic acid production over oceans.

## Results

### Experimental

**Fig. 1:**
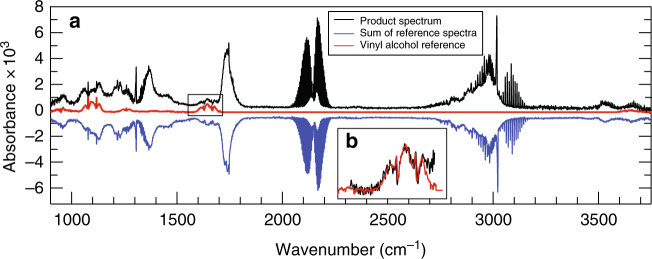
Fourier transform infrared spectra of the photolysis products. **a** Infrared spectrum (black) following 7 min of 315 nm irradiation of a mixture of 10 Torr acetaldehyde in 750 Torr nitrogen. The remaining acetaldehyde has been subtracted from the spectrum. The inverted blue trace shows the sum of scaled reference spectra of photochemical products. The red trace is an infrared reference spectrum of vinyl alcohol, a portion of which, between 1550 and 1700 cm^–1^, is expanded in the inset **b**

Acetaldehyde (AC) at a pressure of 1–10 Torr, mixed with 0–750 Torr of N_2_, was placed in a static Teflon-coated gas cell. The mixture was irradiated with a tunable, pulsed laser at wavelengths in the actinic UV range of *λ* = 300–330 nm (AC does not absorb significantly for *λ* > 330 nm). We detected primary and secondary photochemical products and the depletion of the precursor using Fourier transform infrared (FTIR) spectroscopy. Sequential infrared spectra were averaged in 30 s periods before, during, and after 7 min of UV irradiation. Figure 1 shows a representative spectrum following 315 nm irradiation at 760 Torr total pressure. The signal due to unreacted AC has been subtracted to yield a spectrum solely due to photochemical reaction products. The amount of subtracted signal provides directly the concentration of AC that has been photolyzed (1–4% across various experiments).

We identify and quantify photolysis products by fitting a library of reference spectra, calibrated and measured on the same apparatus, to the spectrum obtained after photolysis of AC. An example of such a fit is shown in Fig. 1 (blue curve). All detected compounds are stable, formed either as direct products of AC irradiation (CO and CH_4_), or from secondary reaction of primary radical products with each other or the parent AC. Vinyl alcohol (VA) is assigned unambiguously by its characteristic spectral features^19^, most prominent near 1100, 1650, and 3650 cm^−1^, as shown in the red reference spectrum in Fig. 1. Other than VA, all other identified products are consistent with the known photochemistry of AC.

The assignments and fitting of reference spectra of all species are described in more detail in Supplementary Note 1. Typical reference spectra used throughout are shown in Supp. Fig. 1. The mass yields (mass of products formed/mass of CH_3_CHO lost) are plotted in Supp. Fig. 2, showing they are close to unity throughout, except at 330 nm. At this wavelength there are unassigned features (Supp. Fig. 3), indicating at least one unidentified product at long wavelengths. Generally, however, we have accounted for all the products from irradiation of AC. All products are stable with respect to time, except for VA itself, which undergoes a wall-catalyzed reverse tautomerization to AC. We record the time-dependence of VA pressure and compensate for this loss as explained in Supp. Note 1 and Supp. Fig. 4.

The quantum yield (QY) of each product was calculated from the known UV absorption cross-section of AC^25^ and the measured photon flux, as described in Methods and Supp. Fig. 5. An example calculation is shown in Supplementary Tables 1 and 2. Attributing each observed product to a primary photolysis event allows us to determine the primary quantum yields for AC irradiation as a function of wavelength and pressure.

The primary photochemistry of AC has been studied in detail because of its importance in atmospheric pathways^25,26^, its role as a prototypical carbonyl^27–29^, and as a target for studies of fundamental new reaction mechanisms^30–32^. There are three primary bond-breaking processes that feature in the near ultraviolet photochemistry of AC:

1 $${\mathrm{CH}}_3{\mathrm{CHO}}\,+\,{{h\nu }}\,\to\,{}^ \bullet {\mathrm{CH}}_3\,+\,{\mathrm{H}}\,{}^ \bullet {\mathrm{CO}},$$

2 $$\to {\mathrm{CH}}_4\,+\,{\mathrm{CO}},$$

3 $$\to {\mathrm{CH}}_3\,{}^ \bullet {\mathrm{CO}}\,+\,\,{}^ \bullet {\mathrm{H}}{\mathrm{.}}$$

**Fig. 2:**
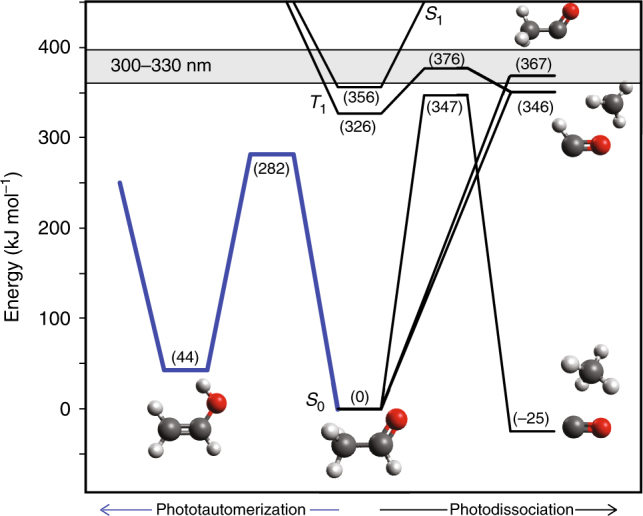
Simplified potential energy diagram. Stationary points describing photochemical pathways of acetaldehyde following excitation at actinic wavelengths of 300–330 nm (gray band). Energies obtained from refs. ^33–37^, isomerization of acetaldehyde to vinyl alcohol indicated in blue

These are shown schematically in Fig. 2, where energies of stable species were obtained from Active Thermochemical Tables^33^, excited state energies from spectroscopic studies^34,35^, and transition state energies from ab initio calculations^36,37^. Absorption of a UV photon in the actinic range excites AC to the lowest lying excited singlet state, *S*_1_, which is bound at these energies. Intersystem crossing (ISC) rapidly populates the lowest triplet state, *T*_1_, where the lowest energy photochemical pathway produces CH_3_ + HCO (Eq. (1)) when *λ* < 318.5 nm^28,38^. At longer wavelength (*λ* > 318.5 nm), a barrier on the *T*_1_ surface prevents dissociation, and AC eventually reaches the ground electronic state, *S*_0_, by sequential ISC (*S*_1_ → *T*_1_ → *S*_0_). Although it is often assumed that internal conversion is responsible for transfer to *S*_0_ in AC, both recent experiments^39^ and theoretical evidence^40^ suggest *S*_1_/*S*_0_ conical intersections are too high in energy to provide a valid pathway at actinic UV energies.

On *S*_0_, there are a number of primary photodissociation processes, including Eqs (1) – (3) above. In addition, AC can readily isomerize prior to dissociation, as evidenced by observation of H/D exchange in photoproducts of selectively deuterated AC^18,37^. The intermediate isomers can be stabilized by collision with a “bath gas”, M. Of particular importance, the keto-enol tautomerization barrier lies well below the photon energy (see Fig. 2) and below any other isomerization barrier. The enol isomer of CH_3_CHO, CH_2_=CHOH, is therefore the most likely isomerization product:

4 $${\mathrm{CH}}_3{\mathrm{CHO}}\,+\,{{h}}\nu\,+\,{\mathrm{M}}\,\to\,{\mathrm{CH}}_2{=}{\mathrm{CHOH }}\,+\,{\mathrm{M}}{\mathrm{.}}$$

**Fig. 3:**
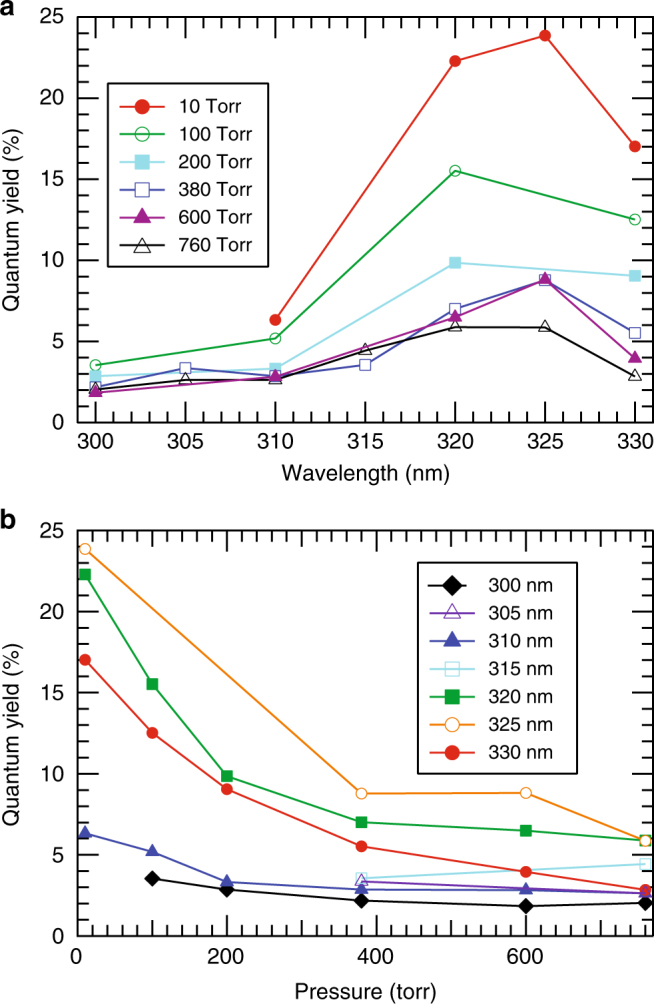
Quantum yields of vinyl alcohol. Quantum yields for vinyl alcohol production as a function of **a** wavelength and **b** pressure at room temperature. Uncertainty ±25% of the stated value

Experimental QYs for VA formation are shown in Fig. 3, and display significant variation with N_2_ pressure and excitation wavelength. At shorter wavelengths (*λ* < 318.5 nm) the *T*_1_ pathway to HCO + CH_3_ dominates (shown in Fig. 2) and the photo-tautomerization QY is small (Fig. 3). Collisions compete with photodissociation at higher pressures, removing internal energy until the molecule has insufficient energy to surmount the *T*_1_ barrier. The total photodissociation QY therefore drops with increasing N_2_ pressure and decreasing photolysis energy. As described in more detail in Supp. Note 1, our results are in good agreement with previous work^25,26^, as shown in Supp. Fig. 6. The QY for VA production, however, generally increases as photolysis energy decreases, with a steeper rise once the kinetically favored dissociation on the *T*_1_ surface becomes energetically inaccessible (Fig. 3). Like the total photodissociation QY, the QY for VA production decreases with increasing pressure, as AC is collisionally quenched below the *S*_0_ barrier to tautomerization.

### Atmospheric modeling

In the troposphere, da Silva calculated that OH will add to either end of the enol C=C double bond with negative activation energy^21^:

5a $${\mathrm{CH}}_2 {=} {\mathrm{CHOH}} + {}^ \bullet {\mathrm{OH}} \to {}^ \bullet {\mathrm{CH}}_2{-}{\mathrm{CH}}\left( {{\mathrm{OH}}} \right)_2,$$

5b $$\to {\mathrm{CH}}_2{\mathrm{OH}}{-}{}^ \bullet {\mathrm{CHOH}},$$

where formation of the α-substituted product (Eq. (5a)) is favored. These radicals react rapidly with O_2_, forming peroxy radicals:

6a $$^ \bullet {\mathrm{CH}}_2{-}{\mathrm{CH}}\left( {{\mathrm{OH}}} \right)_2 + {\mathrm{O}}_2 \to \,^ \bullet {\mathrm{OOCH}}_2{-}{\mathrm{CH}}\left( {{\mathrm{OH}}} \right)_2,$$

6b $${\mathrm{CH}}_2{\mathrm{OH}}{-}{}^ \bullet {\mathrm{CHOH}} + {\mathrm{O}}_2 \to {\mathrm{CH}}_2{\mathrm{OH}}{-}{\mathrm{C}}\left( {{\mathrm{OO}}^ \bullet } \right){\mathrm{HOH}}{\mathrm{.}}$$

The peroxy radical from (Eq. (6a)) undergoes a 1,5 H-shift followed by decomposition to OH + CH_2_O + HCOOH, whereas the radical from (Eq. (6b)) dissociates to gylcolaldehyde:

7a $$^ \bullet {\mathrm{OOCH}}_2-	{\mathrm{CH}}\left( {{\mathrm{OH}}} \right)_2 \to {\mathrm{HOOCH}}_2-{\mathrm{C}}\left( {{\mathrm{O}}^ \bullet } \right){\mathrm{HOH}} \\ \to ^ \bullet {\mathrm{OH}} +	 {\mathrm{CH}}_2{\mathrm{O}} + {\mathrm{HCOOH}},$$

7b $${\mathrm{CH}}_2{\mathrm{OH}}{-}{\mathrm{C}}\left( {{\mathrm{OO}}{}^ \bullet } \right){\mathrm{HOH}} \to {\mathrm{CH}}_2{\mathrm{OH}} {-} {\mathrm{CHO}}{\mathrm{ + HOO}}^ \bullet {\mathrm{.}}$$

To test the importance of AC photo-tautomerization in the atmosphere, we need to consider both the detailed atmospheric chemistry, as well as global transport mechanisms. We have used a complementary approach, incorporating the quantum yields measured in this work (Fig. 3), along with Eqs (5a)–(7b), into two atmospheric models: a zero-dimensional atmospheric chemistry box model utilizing the Master Chemical Mechanism (MCM) version 3.2^23^, and a global chemical transport model (GEOS-Chem 3D CTM)^24^. The first is state-of-the-art in terms of chemical complexity, but as a box model lacks transport effects and regional or global context. The second provides fully resolved transport and global context but as a global model cannot be as chemically explicit.

Two MCM box model simulations were run to test the importance of photo-tautomerization in clean and polluted regions of the atmosphere. The first simulation was constrained with gas-phase data, including observed AC mixing ratios, photolysis rates, and meteorological parameters from the 2007 RHaMBLe project at the Cape Verde Atmospheric Observatory^41^. The second simulation was constrained with field data from the summer 2012 ClearfLo project^42^ that took place at an urban background site in London. At Cape Verde, HCOOH was detected with a reported median value of 128 pptv^43^; in London concentrations were much higher, with a peak concentration of 6.7 ppbv^44^.

In both simulations, the time-dependent photo-tautomerization rate was calculated by integrating the product of the AC absorption cross-section^45^, the 760 Torr experimental quantum yield (this work) and the time-dependent photon flux as a function of wavelength. We used da Silva’s total rate coefficient for pathway 5a + 6a of 3.8×10^−^^11^ cm^3^ molecule^−^^1^ s^−^^1^^ 21^.

We also investigated the impact of the recently reported acid-catalyzed gas-phase reverse tautomerization of VA back to AC. Two additional simulations were run with the two acid-catalyzed gas-phase reverse tautomerization rate coefficients (i) 1.3×10^−14^ cm^3^ molecule^−1^ s^−1^ at 298 K as used in ref. ^21^ or (ii) 2.0×10^−20^ cm^3^ molecule^−1^ s^−1^ as reported in ref. ^22^. Using the larger rate coefficient for gas-phase reverse tautomerization, the modeled FA concentrations were reduced by ~6% in both clean and polluted model scenarios whereas the smaller rate coefficient had no observed effect; that is, we conclude gas-phase reverse tautomerization had no observed effect on FA yields.

**Fig. 4:**
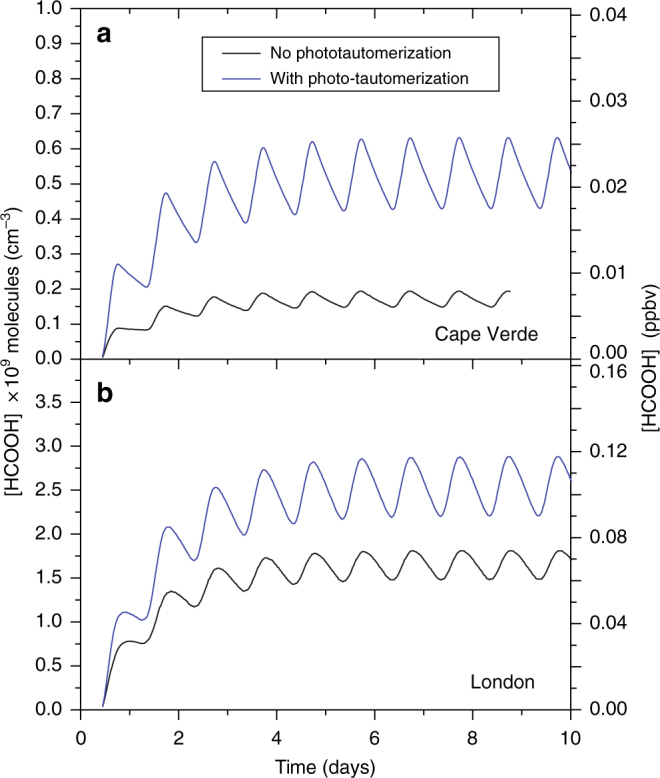
Master Chemical Mechanism simulations. Master Chemical Mechanism box model simulations of formic acid production with (blue) and without (black) photo-tautomerization and OH + vinyl alcohol reactions. Two distinct atmospheric conditions are shown: **a** pristine, ocean environment at Cape Verde, Sao Vincente Island, and **b** urban environment at London, UK

The application of the MCM to clean air and polluted conditions is shown in Fig. 4, with and without the photo-tautomerization mechanism. Including photo-tautomerization in the MCM gives a factor of ~1.7 increase in FA in London, and a factor of ~3 increase in Cape Verde, showing that this mechanism represents a major change relative to the known photochemical sources of FA in these environments. However, we also see in Fig. 4 that it is still not enough to explain the observed concentration of FA in these two locations. The MCM box model considers only photochemical sources of FA, while omitting direct emissions and any transport effects that may also contribute to the observed concentrations. We therefore apply the GEOS-Chem 3D global CTM to assess the global impact of the photo-tautomerization mechanism in a manner that accounts for not only the photochemical production and loss of FA, but also non-photochemical sources, transport, and deposition.

**Fig. 5:**
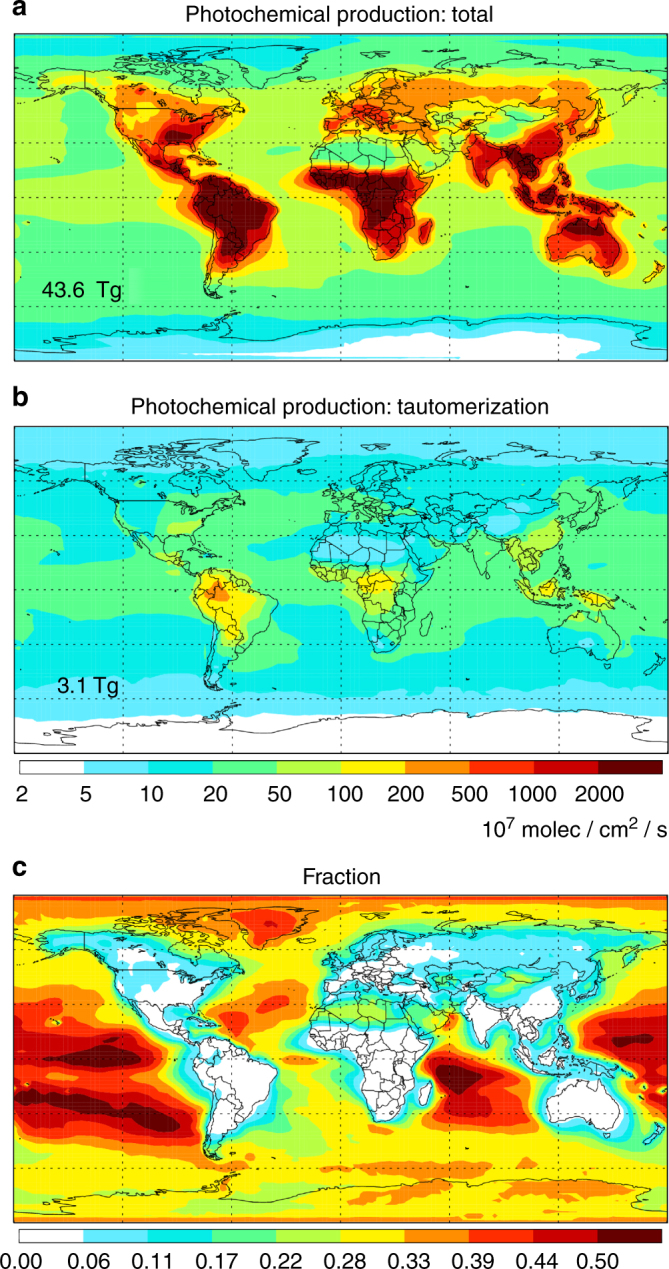
Column-integrated formic acid (FA) production rates as simulated by the GEOS-Chem 3D chemical transport model as a function of latitude and longitude. **a** Total FA production from all chemical pathways. **b** FA produced via photo-tautomerization of AC. **c** Fraction of total FA produced via photo-tautomerization

Figure 5 shows the column-integrated FA production rates from photo-tautomerization and from all photochemical sources combined as simulated by GEOS-Chem (Supp. Fig. 7 shows and Supplementary Note 2 discusses the results separately for the lower- and free-troposphere). Previous application of the GEOS-Chem 3D model used a single estimated photo-tautomerization quantum yield and an overestimate of the gas-phase reverse tautomerization rate coefficient^10^. Here we use our experimental pressure- and wavelength-dependent quantum yields and more realistic rate coefficients to better estimate the importance of the photo-tautomerization of AC. Using this data, we predict the photo-tautomerization pathway produces 3.1 Tg/y of FA, approximately 7% of the total simulated photochemical sources. As shown in Fig. 5, the fractional contribution of photo-tautomerization to FA production is highest over remote areas and is the dominant model source over many ocean regions. For example, Fig. 5(c) reveals that photo-tautomerization accounts for 40–60% of the column-integrated photochemical FA production in GEOS-Chem over a large part of the global oceans. FA and other carboxylic acids have long been known to be ubiquitous in the remote marine boundary layer, with uncertain origin^1,46^, where they can affect aqueous-phase S^IV^ oxidation on marine aerosols^47^. The simulations here show that marine emissions of AC and its precursors^16,48^ dominate the modeled FA production in such regions. Conversely, the relative contribution of photo-tautomerization to FA production is small over continental regions where isoprene and other terrestrially emitted VOCs dominate as FA sources.

Although photo-tautomerization of AC does provide a source of FA, it alone cannot account for the factor of two or more discrepancy seen between experimental and modeled FA concentrations worldwide^10,16^. However, the role of photo-tautomerization of other carbonyls has not been explored. For example, the known photochemistry of propanal^49,50^ and acetone^51–53^ is broadly similar to AC, with similar tautomerization barrier heights^54^. We might therefore expect photo-tautomerization efficiencies to be broadly similar in these carbonyls under atmospheric conditions. There are also a large number of short and branched chain carbonyls in the atmosphere although larger carbonyls, such as butanal^55^, have other photochemical pathways, e.g. the Norrish Type II mechanism which also yields enols. The relative rates and quantum yields of these processes, in comparison to tautomerization, are unknown; tautomerization kinetics should be evaluated to characterize possible formation mechanisms of larger organic acids.

Although more work on sources and sinks of enols in the atmosphere is warranted, the present results argue strongly that photo-tautomerization of carbonyl molecules plays a key role in organic acid production in Earth’s atmosphere that must be included in quantitative models.

## Methods

### Laser photolysis Fourier transform infrared spectroscopy

Pre-mixed acetaldehyde vapor and N_2_ gas were flowed into a Teflon-coated stainless steel gas cell using two calibrated mass flow control valves (MKS). The total pressure was measured using a calibrated capacitance manometer (MKS). The partial pressure of AC was confirmed in situ by recording an FTIR spectrum before photolysis and comparing to a reference spectrum. Gas mixtures were prepared in the range 1–20 Torr neat AC, and 10 or 20 Torr AC mixed with approximately 50, 100, 200, 380, 600, and 750 Torr N_2_.

The static gas mix was irradiated at wavelengths between 300 and 330 nm by the frequency doubled output of an OPO, pumped by a Nd:YAG laser. Typical laser energy was 3–6 mJ pulse^−1^. The laser beam was reflected through the cell four times. Laser energy was measured for each pass of the laser using an energy meter. These measurements, when taken with an empty cell, provided the losses for each optical component (mirrors and windows). When AC was admitted to the cell, the output laser energy dropped due to absorption of the laser radiation by AC, from which the number of photons absorbed can be derived. From the known path length and pressure of acetaldehyde, the absorption cross-section was calculated, which was in good agreement (±10%) with the literature value^25^.

FTIR spectra at 1 cm^−1^ resolution were recorded before photolysis and continuously during irradiation for 5–7 min. Spectra were saved every 30 s. FTIR spectra were recorded for an additional 5–7 min after laser irradiation was halted. The time profile of all stable products showed a linear rise in absorption with irradiation time, followed by constant absorption after irradiation ceased. VA, however, showed a non-linear rise in absorption, followed by a decay in intensity for up to 15 min, as shown in Supplementary Note 1, Supp. Figure 4. Measurement of the decay time provided the wall reaction rate and convolving with a linear rise provided the rate of production and hence the QY in absence of wall reactions. Measurements were acquired over three laboratory sessions and repeat measurements were acquired both within and between sessions. Reproducibility between the resulting spectra was very good, yielding an estimated uncertainty in the final quantum yields of ±25%.

### Atmospheric modeling

The GEOS-Chem 3D model runs employed meteorological data from the GEOS-5 Forward Processing (GEOS-FP) Atmospheric Data Assimilation System, which have a native resolution of 0.25° latitude×0.3125° longitude and 72 vertical levels. For the present analysis we degraded the resolution to 2°×2.5° with 47 vertical levels and used a 15-min transport time step and 1-year model spinup. The model includes detailed HO_x_-NO_x_-VOC-O_3_-aerosol tropospheric chemistry, air-sea exchange, and deposition (wet + dry), with extensive updates for the simulation of FA as described in ref. ^10^. Further model details relevant to the simulations shown here (emissions, other chemical pathways leading to FA) are provided by Millet et al.^10^.

### Data availability

The data that support the findings of this study are available from the authors on reasonable request; see author contributions for specific data sets.

## Electronic supplementary material

Supplementary Information
