# Supplementary material for: Photo-tautomerization of acetaldehyde as a photochemical source of formic acid in the troposphere
Source: Nat Commun. 2018 Jul 3;9:2584. doi: 10.1038/s41467-018-04824-2 (PMC6030138; doi:10.1038/s41467-018-04824-2)
Supplement: Supplementary file 1 — Supplementary Information [file 41467_2018_4824_MOESM1_ESM.docx]

Cover Page

**Photo-tautomerization of acetaldehyde as a photochemical source of formic acid in the troposphere**

Shaw et al.

**Photo-tautomerization of acetaldehyde is a significant photochemical source of formic acid in the troposphere**

Miranda Shaw,^1^ Balint Sztaray,^2^ Lisa K. Whalley,^3^ Dwayne E. Heard,^3^

Dylan B. Millet,^5^ Meredith J. T. Jordan,^1^ David L. Osborn,^4^* and Scott H. Kable^6^*

1. *School of Chemistry, University of Sydney, New South Wales 2006, Australia*

*Department of Chemistry, University of the Pacific, Stockton, California 95211, USA*

*School of Chemistry and National Centre for Atmospheric Science, University of Leeds, Leeds, LS2 9JT, United Kingdom*

*Combustion Research Facility, Sandia National Laboratories, Livermore, California 94551, USA*

*Department of Soil, Water, and Climate, University of Minnesota, Minneapolis–Saint Paul, MN 55108, USA*

*School of Chemistry, University of New South Wales, New South Wales 2052, Australia*

* Corresponding authors: s.kable@unsw.edu.au; dlosbor@sandia.gov

Supplementary Information

**Supplementary Note 1**

This Supplementary Note provides further information about the spectroscopic assignments in the FTIR spectrum and the methodology for determining quantum yields.

**Assignment of the FTIR spectrum**

Supplementary Figure 1 shows a representative experimental spectrum (red) along with each individual fitted spectrum of the identified products (black), the sum of these (green), and the residual (blue). This spectrum was taken after 7 minutes irradiation at 315 nm of 10 Torr of acetaldehyde and 750 Torr of N_2_. The residual spectrum shows some features that arise due to saturation by the parent acetaldehyde (particularly in the CO stretching region near 1750 cm^−1^), and a trace amount of unidentified product at 1000 and 1150 cm^−1^.

**Mass yields**

Mass yields for each identified product were calculated for each experiment by the ratio of mass of product formed to mass of acetaldehyde consumed, where the changes in mass were found by fitting reference spectra of known number density to the experimental spectra.

The total mass yields were 100±15% for the majority of wavelengths and pressures, as shown in Fig. S2. The associated error is due to the difficulty in fitting the multiple overlapping reference spectra, particularly for the heavier secondary products, which have relatively low quantum yields, but make a large contribution to the mass yield.

At the longest wavelengths, the total mass yield drops to ~60-80%. Supplementary Figure 3 shows the FTIR spectrum, sum of reference spectra, and residual spectrum for photolysis at 330 nm and 760 torr total pressure. The sum of reference spectra still provides an excellent fit to the observed spectrum and the VA signature is still clear and unambiguous. However, there are now a small number of unassigned features, for example, near 3200-3300 cm^–1^ is a broad feature that is likely to be an OH stretch of an unidentified product, and near 1100 cm^–1^ are a number of weak, broad and complex features that are unassigned.

As the photon energy is lowered below the energy of the triplet barrier (corresponding to 320 nm), the photochemistry becomes dominated by reaction on the ground state potential energy surface, including photo-tautomerization. These hot products seem to undergo a variety of more complex bimolecular reactions, forming larger molecules such as 3-hydroxybutanone and biacetyl. The remaining mass yield is therefore likely to be comprised of a small mole fraction of one or more higher molecular weight species. The IR spectrum might overlap existing features, or these higher molecular weight products might stick to the walls of the cell. Irrespective, this is unlikely to affect the measured quantum yield of vinyl alcohol.

Correction for wall-catalyzed VA → acetaldehyde tautomerization

Vinyl alcohol (VA) is produced via the unimolecular reaction (i) with rate constant *k_p_*:

CH_3_CHO + hν $\underset{\to}{kp}$ CH_2_=CHOH (i)

As only 2-4% of the acetaldehyde is photolysed, the number density of acetaldehyde can be approximated as constant, so reaction (i) can be considered to have pseudo-zeroth order kinetics, with an effective rate constant of *k_p_’*:

*k_p_’* = *k_p_*[acetaldehyde] (ii)

Although produced at a constant rate, the observed number density of vinyl alcohol does not increase linearly as it also undergoes wall-catalyzed, first order decay back to acetaldehyde, with rate constant *k_d_*:

CH_2_=CHOH $\underset{\to}{kd}$ CH_3_CHO (iii)

The overall production and decay of vinyl alcohol can therefore be described by the rate equation (iv):

$\frac{d}{dt}$[VA] = *k_p_’* – *k_d_*[VA] (iv)

The value of the decay constant, *k_d_*, was found by observing the change in number density of VA in the minutes following irradiation, and fitting these to a first order exponential decay equation:

[VA] = [VA]_t=0_$e^{-k_{d}t}$ (v)

where *t*=0 corresponds to the end of irradiation.

The value for *k_p_’* was found by incorporating the determined value for *k_d_* into the integrated form of equation (iv), and fitting the resulting equation to the observed number density of VA during the 7 minutes of irradiation, as shown in Fig. S4.

[VA] = $\frac{k_{p}’}{k_{d}} [1-e^{-k_{d}t}]$ (vi)

The ideal, linear change of VA number density, in the absence of wall-catalyzed loss, is therefore found by equation (vii):

[VA] = (*k_p_’*)*t* (vii)

Both *k_p_’* and *k_d_* are dependent on the experimental conditions, such as photolysis wavelength, laser power, and cell coating, so this process was performed for all experimental data sets. The ideal VA number density from equation (vii) was typically around 20-40% higher than the observed VA number density, and this correction was applied to the final quantum yields. Stable products arising from the photodissociation channels, such as CO and methane, showed the expected linear growth during irradiation, with no significant change in number density in the time following.

*Quantum yield calculations*

Quantum yields of each identified product were calculated as the number density (molecules/cm^3^) of a product produced divided by the number density of electronically excited acetaldehyde generated over 7 minutes of irradiation. The former was determined by fitting the FTIR spectrum, as described previously. The number density of excited acetaldehyde molecules was found by monitoring the laser energy output (in mJ/pulse), and using the Beer-Lambert law to calculate the amount of energy absorbed during each pass of the laser beam.

A schematic of the cell and laser path is shown in Supplementary Figure 5. The amount of energy lost at each window and mirror surface was determined separately for each wavelength by measuring the laser power before and after each surface, in the absence of any sample.

The total energy absorbed in Joules was converted to a number of photons absorbed, and assumed to be equal to the number of molecules of acetaldehyde that were excited. This assumption is based on the small overall decrease in acetaldehyde number density (2-4%), and the corresponding small increase in population of other UV-absorbing products. Explicitly taking these changes into account didn’t change the final yields by any significant amount.

An example calculation is presented in Supplementary Tables 1 and 2 below. Note that the calculation begins from the end of the laser path and works backwards.

*Photodissociation quantum yields and comparison with literature*

Acetaldehyde has three photodissociation channels that result in cleavage of the C-C bond:

CH_3_CHO($T_{1}^{*}$) →•CH_3_ + H•CO (viii)

CH_3_CHO($S_{0}^{*}$) →•CH_3_ + H•CO (ix)

CH_3_CHO($S_{0}^{*}$) → CH_4_ + CO (x)

The methyl and formyl radicals subsequently undergo the following reactions to form stable species:

•CH_3_ + H•CO → CH_4_ + CO (xi)

•CH_3_ + •CH_3_ +M → C_2_H_6_ + M (xii)

•CH_3_ + CH_3_CHO → CH_4_ + CH_3_•CO (xiii)

H•CO + H•CO → CO + H_2_CO (xiv)

H•CO + CH_3_•CO + M → CH_3_COCHO + M (xv)

The total yield of photodissociation reactions (viii), (ix) and (x) can therefore be found by the sum of the yields of either the carbonyl containing, or methyl containing end products, as observed by FTIR. The yields shown in Supp. Fig. 6 are the average of these two measures.

Our photolysis yields are broadly consistent with those found by Horowitz and Calvert (1) and Moortgat *et al.* (2) although slightly lower at short wavelengths and higher at long wavelengths. This trend was also observed in our previously reported, low pressure acetaldehyde experiments. (3)


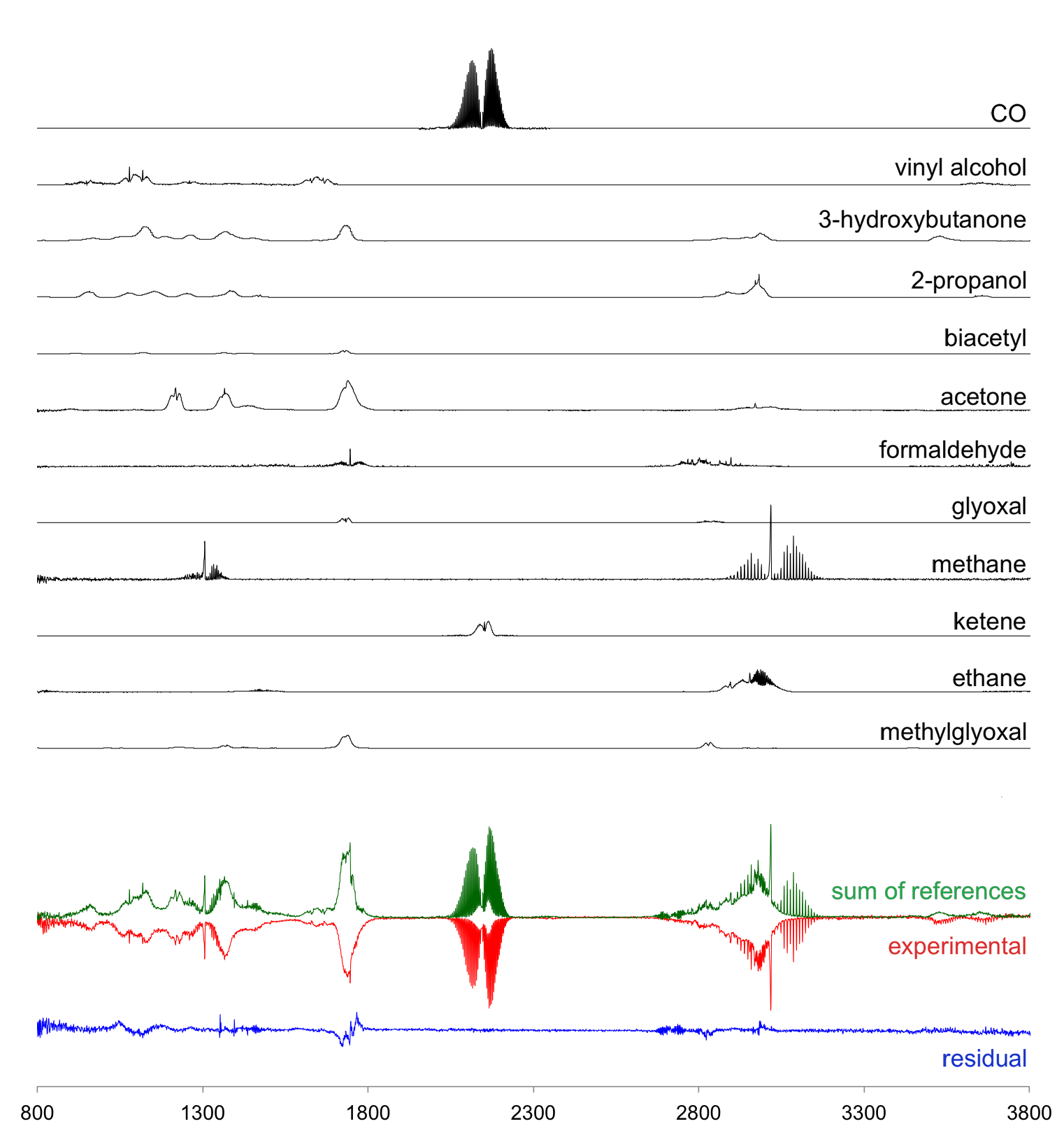


Supplementary Figure 1 Experimental and fitted reference spectra for photolysis of acetaldehyde at 315 nm.


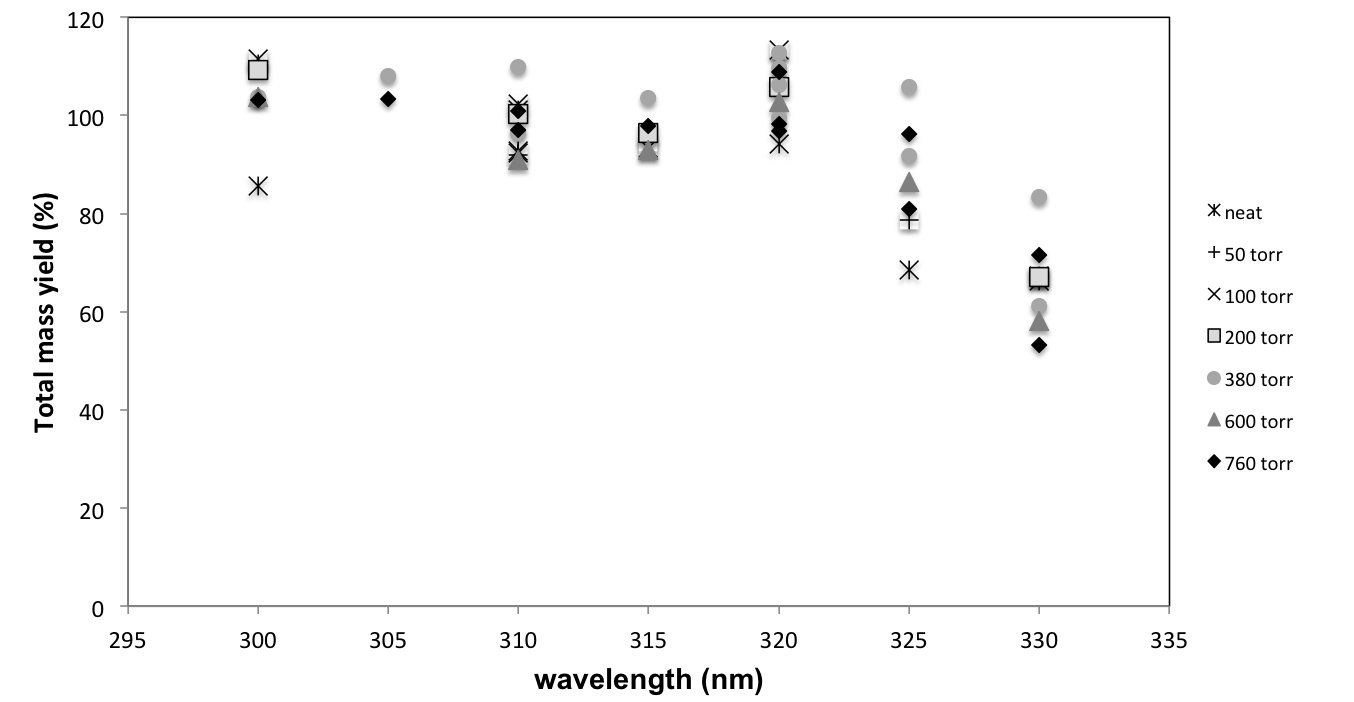


Supplementary Figure 2 Mass yields for all experiments (10 Torr acetaldehyde, with 0-750 Torr of N_2_).


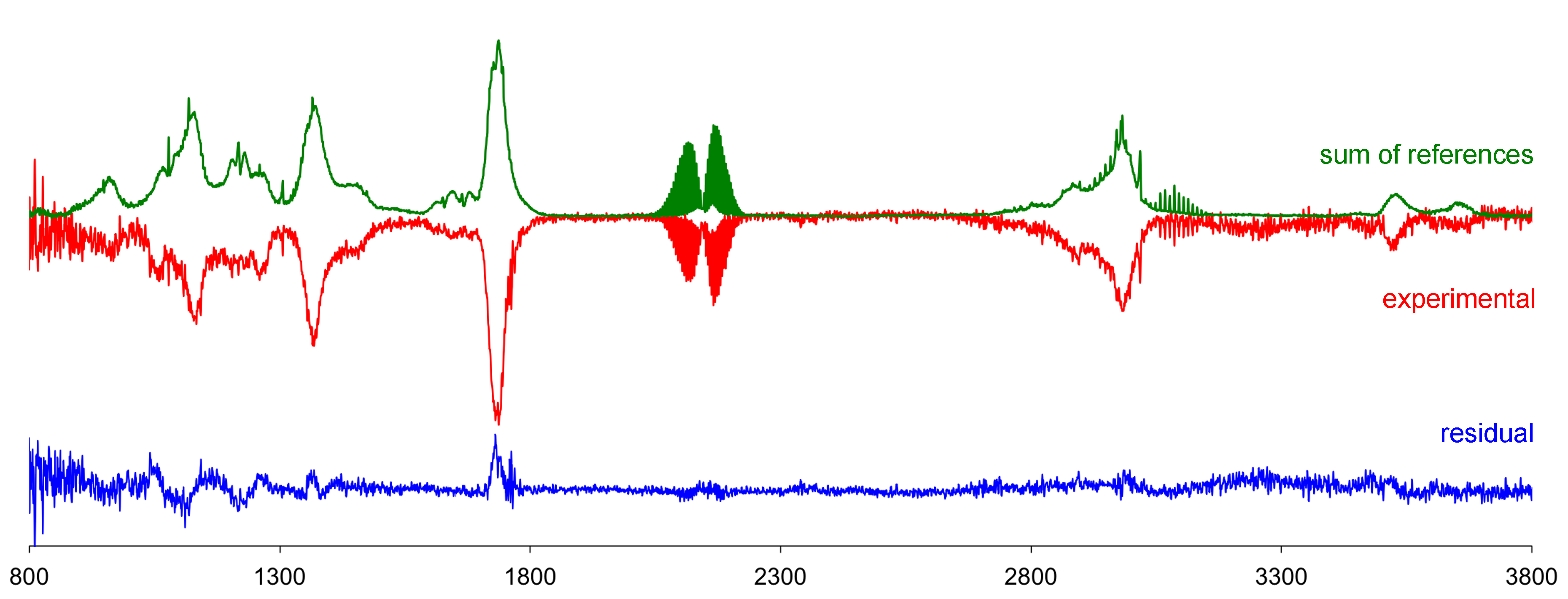


Supplementary Figure 3 Experimental and fitted reference spectra for photolysis at 330 nm of 10 Torr acetaldehyde and 750 Torr N_2_. A small number of spectral features are unassigned and likely responsible for the lower mass yield accounted for at this wavelength (see Supp. Fig. 2).


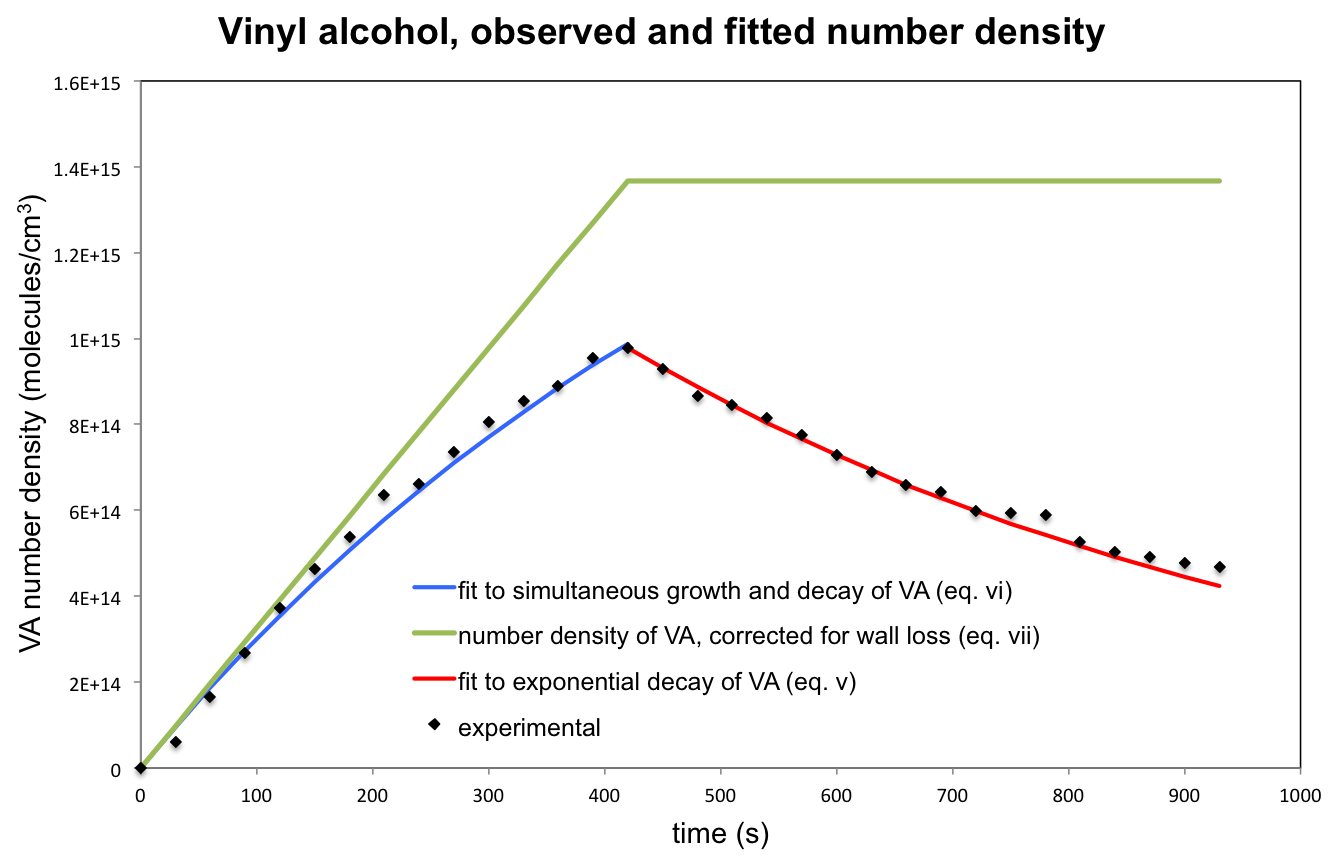


Supplementary Figure 4 Vinyl alcohol decay kinetics. Example of the growth and decay of vinyl alcohol over time, following 7 minutes irradiation of 10 Torr of acetaldehyde at 320 nm.


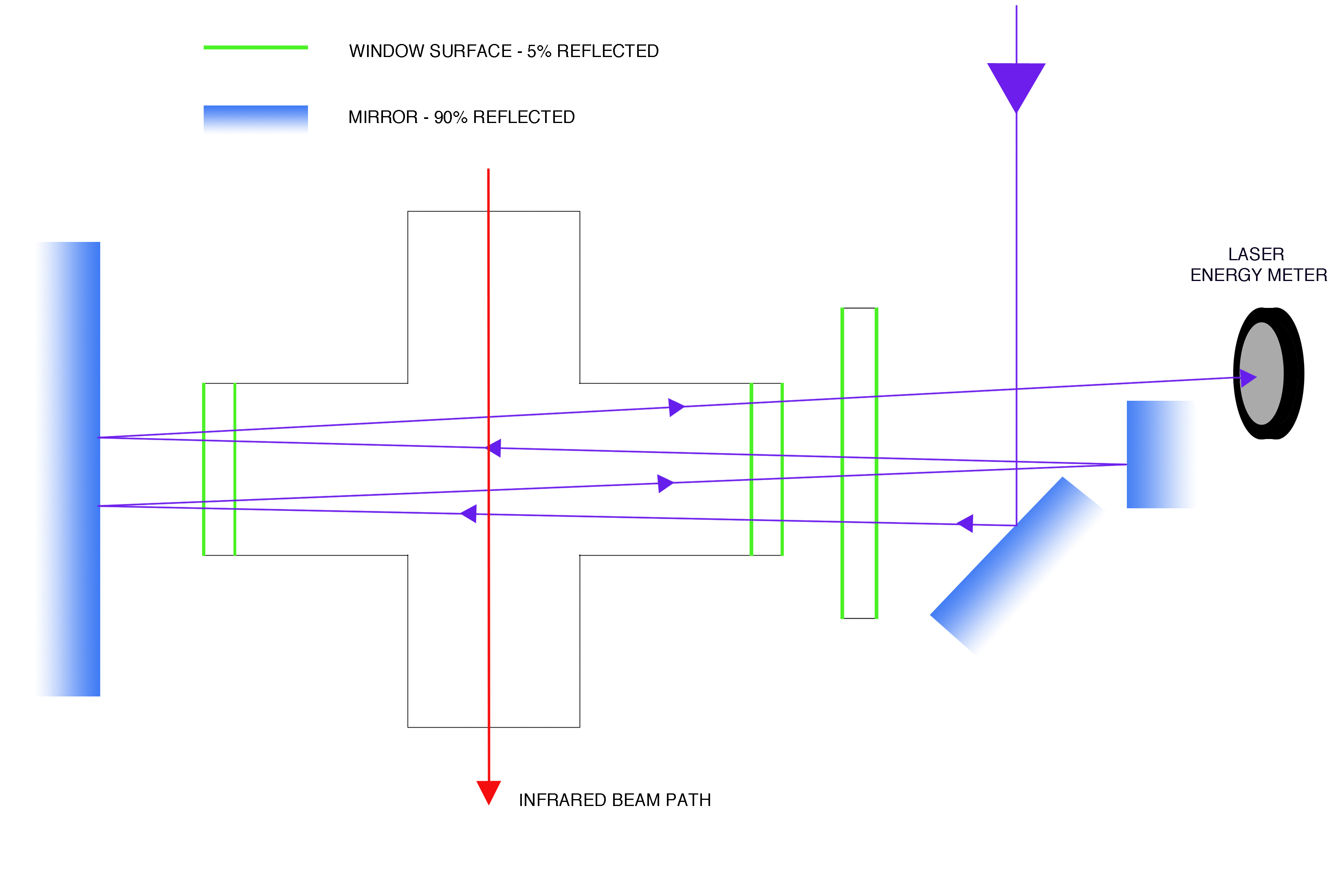


Supplementary Figure 5 Schematic of the experimental setup.

**Supplementary Table 1** Miscellaneous data for Quantum Yield Calculations.

| **Laser** |  |  |  |
| --- | --- | --- | --- |
| Pulse energy | *E*_f_ | 1.0 | mJ pulse^−1^ |
| Wavelength | λ | 300 | nm |
| Energy per photon | *h*c/$\lambda$ | 6.62E−19 | J photon^−1^ |
| Frequency | *f* | 10 | pulse s^−1^ |
| **Acetaldehyde sample** |  |  |  |
| Pressure | *P* | 10 | Torr |
| Number density | *N* | 3.24E+17 | molec cm^−3^ |
| Cross section | σ | 4.16E−20 | molec^−1^ cm^2^ |
| **Cell and Experiment** |  |  |  |
| Cell volume | *V* | 191.6 | cm^3^ |
| Path length | *L* | 12.4 | cm |
| % T at windows | *T*_w_ | 95% |  |
| % T at mirrors | *T*_m_ | 88% |  |
| Time | *t* | 420 | s |

Fraction of light transmitted in each laser pass (Beer’s Law): $\frac{E_{out}}{E_{in}}=e^{-Nl\sigma}=0.846$

**Supplementary Table 2** Example Quantum Yield Calculation.

|  |  |  | E (mJ.pulse^−1^) | |
| --- | --- | --- | --- | --- |
| E at final point | *E*_f_ | (measured directly) | 1.00 |  |
| E immediately after 4th pass | *E*4_out_ | = *E*_f_/(*T*_w_)^4^ | 1.23 |  |
| E immediately before 4th pass | *E*4_in_ | = *E*4_out_/0.846 | 1.45 |  |
| E absorbed during 4th pass | *A*4 | = *E*4_in_ − *E*4_out_ | 0.22 |  |
| E immediately after 3rd pass | *E*3_out_ | = *E*4_in_/(*T*_m_.*T*_w_^4^) | 2.02 |  |
| E immediately before 3rd pass | *E*3_in_ | = *E*3_out_/0.846 | 2.39 |  |
| E absorbed during 3rd pass | *A*3 | = *E*3_in_− *E*3_out_ | 0.37 |  |
| E immediately after 2nd pass | *E*2_out_ | = *E*3_in_/(*T*_m_.*T*_w_^8^) | 4.10 |  |
| E immediately before 2nd pass | *E*2_in_ | = *E*2_out_/0.846 | 4.84 |  |
| E absorbed during 2nd pass | *A*2 | = *E*2_in_ − *E*2_out_ | 0.75 |  |
| E immediately after 1st pass | *E*1_out_ | = *E*2_in_/(*T*_m_.*T*_w_^4^) | 6.76 |  |
| E immediately before 1st pass | *E*1_in_ | = *E*1_out_/0.846 | 7.99 |  |
| E absorbed during 1st pass | *A*1 | = *E*1_in_ − *E*1_out_ | 1.23 |  |
|  |  |  |  |  |
| Sum of E absorbed (per pulse) | *A*_sum_ | = *A*1 + *A*2 + *A*3 + *A*4 | 2.57 | mJ pulse^−1^ |
|  |  |  |  |  |
| Total E absorbed over 7 minutes | *A*_tot_ | = *A*_sum_(*f*)(*t*) | 10.8 | J |
|  |  |  |  |  |
| Total number of photons absorbed | ph_tot_ | = *A*_tot_/(*h*c/$\lambda$) | 1.63E+19 | photons |
|  |  |  |  |  |
| Number density of excited AC | *N*_ex_ | = ph_tot_/*V* | 8.50E+16 | molec cm^−3^ |
|  |  |  |  |  |
| Number density of VA | *N*_VA_ | (from FTIR) | 3.00E+15 | molec cm^−3^ |
|  |  |  |  |  |
| Quantum yield of VA | $\Phi$_VA_ | = N_VA_/N_ex_ | 4% |  |


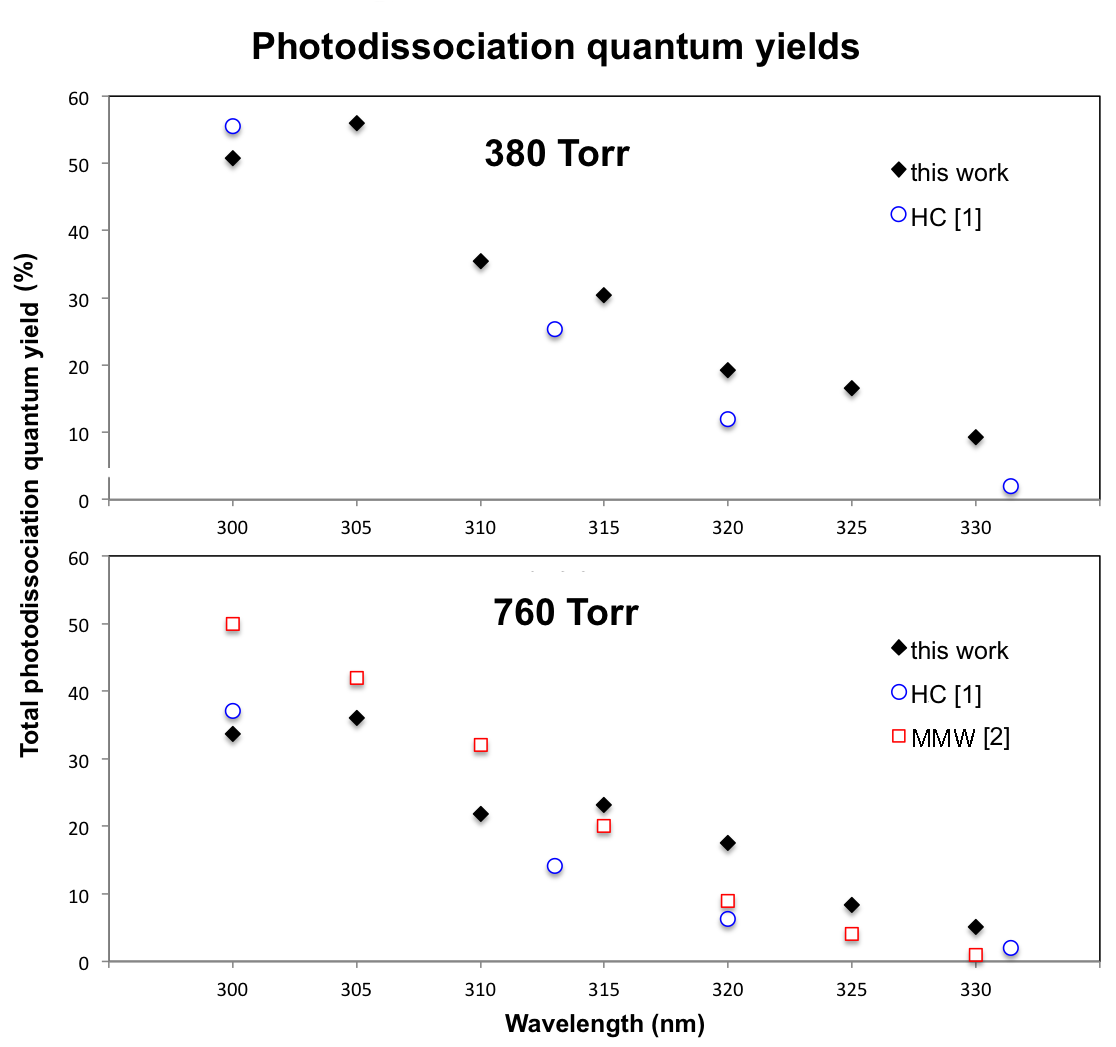


Supplementary Figure 6 Total photodissociation quantum yields. Results from photolysis experiments with 10 Torr acetaldehyde and 370 or 750 Torr of N_2_ in comparison with previous literature results. HC = Horowitz and Calvert (1). MMW = Moortgat, Meyrahn and Warneck (2).

**Supplementary Note 2**

This Supplementary Note provides further information about the GEOS-Chem 3D simulation results for formic acid production as a function of altitude.

Supplementary Figure 7 shows the global distribution of formic acid sources from a GEOS-Chem 3D simulation, as described in the main text and in Ref. (4). This figure is analogous to Fig. 5 in the main text but has been determined for two altitude ranges, corresponding to the lower and free troposphere.

In the free troposphere, photo-tautomerization gives a global formic acid background of 1-10 ppt and is fractionally most important at higher latitudes. Continental outflow of anthropogenic and biogenic volatile organic compounds (and their oxidation products) predominate at lower latitudes. The column-integrated formic acid distribution, Fig. 5 in the main text, is very similar to that obtained near the Earth’s surface, with photo-tautomerization providing the largest formic acid source in many parts of the global marine boundary layer.


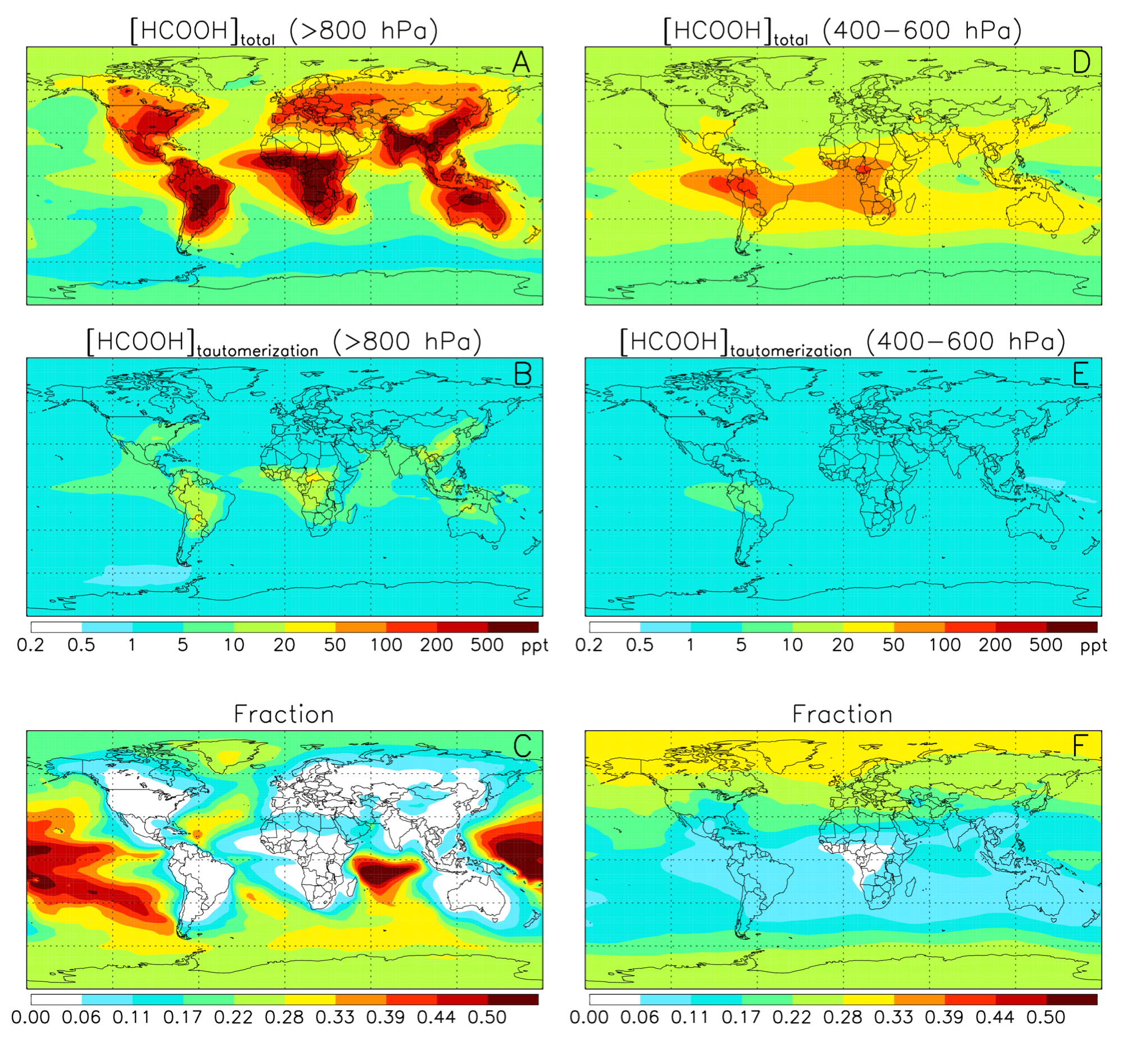


**Supplementary Figure 7** GEOS-Chem 3D model results. Photochemical production of formic acid as a function of altitude, latitude and longitude, as simulated by the GEOS-Chem CTM. Panels A-C show results for the lower troposphere (>800 hPa), while panels D-F show results for the free troposphere (400-600 hPa). Upper panels: total formic acid production; middle panels: formic acid produced via the photo-tautomerization of acetaldehyde; lower panels: fraction of total formic acid produced via photo-tautomerization.

**Supplementary References**

(1) Horowitz, C. J.; Calvert, J. G., Wavelength dependence on the primary processes of acetaldehyde photolysis, *J. Phys. Chem.* **86**, 3105–3114 (1982).

(2) Moortgat, G. K.; Meyrahn, H.; Warneck, P., Photolysis of acetaldehyde in air: CH_4_, CO and CO_2_ quantum yields, *ChemPhysChem,* **11**, 3896-3908 (2010).

(3) Clubb, A. E.; Jordan, M. J. T.; Kable, S. H.; Osborn, D. L., Phototautomerization of acetaldehyde to vinyl alcohol: A primary process in UV-irradiated acetaldehyde from 295 to 335 nm, *J. Phys. Chem. Lett.* **3**, 3522-3526 (2012).

(4) Millet, D. B., *et al.*, A large and ubiquitous source of atmospheric formic acid. *Atmos. Chem. Phys*. **15,** 6283–6304 (2015).
